# Synthesis of New Pentacyclo[5.4.0.0^2,6^.0^3,10^.0^5,9^]undecane-8,11-dione (PCU) Cyanosilylated Derivatives Using Sulphated Zirconia and Hydrotalcite as Catalysts in Microwave-Assisted Reactions under Solvent Free Conditions

**DOI:** 10.3390/molecules16086561

**Published:** 2011-08-04

**Authors:** Nahí Adriana Guerra-Navarro, Laura Nadxieli Palacios-Grijalva, Deyanira Angeles-Beltrán, Guillermo E. Negrón-Silva, Leticia Lomas-Romero, Eduardo González-Zamora, Rubén Gaviño-Ramírez, Juan Navarrete-Bolaños

**Affiliations:** 1 Department of Basic Sciences, UAM-A, Av. San Pablo, No. 180. C.P. 02200, México, D.F., Mexico; 2 Department of Chemistry, UAM-I, San Rafael Atlixco, No. 186. C.P. 09340, México, D.F., Mexico; 3 Institute of Chemistry, UNAM, Circuito Exterior C.U., C.P.04515, México, D.F., Mexico; 4 IMP, Eje Central Lázaro Cárdenas No. 152 San Bartolo Atepehuacan, C.P. 07730 México, D.F., Mexico

**Keywords:** sulphated zirconia, hydrotalcite, catalysts, PCU, microwaves

## Abstract

A comparison was made of the effectiveness of the functionalization reactions of pentacyclo[5.4.0.0^2,6^.0^3,10^.0^5,9^]undecane-8,11-dione (PCU) using sulphated zirconia in protection-deprotection reactions and Mg/Al hydrotalcite in a cyanosilylation reaction, under classical thermal conditions and imposing microwave radiation; improved yields and reaction times were considered.

## 1. Introduction

Organic transformations of polycyclic cage compounds has been the aim of several research groups since the results of Cookson and coworkers were published in 1958 [1] The synthesis of such structures involves functionalization of pentacyclic [5.4.0.0^2,6^.0^3,10^.0^5,9^]undecane-8,11-dione based on the protection of one of its reactive centers. This type of reaction is reported using benzene as solvent and *p*-toluenesulfonic acid as catalyst to afford the desired product in a 74% yield after a reaction time of 4 days [2]. Manipulation of protective groups plays an important role in organic chemistry since this avoids interacting with reactive centers that cannot be involved in certain reactions, so it is necessary to carry out protection and deprotection reactions with high yields [3].

Various models have been proposed to explain the surface structure and nature of catalytically active sites in sulphated zirconia (SZ) [4,5,6,7,8], most of which take into account the formation of Lewis as well as Brønsted acid sites. Formation of Lewis acid sites may be ascribed to the highly covalent character of the adsorbed sulphates and formation of Brønsted sites result from the interaction of water molecules with these sulphates [6,9]. Sulphated zirconia is an excellent eco-friendly heterogeneous catalyst in organic reactions [7,10,11,12]. Hydrotalcites, a family of anionic clays, are known as layered double hydroxides (LDH) are an important class of heterogeneous catalysts in organic transformation [13,14,15,16]. A salient feature of as-synthesized or uncalcined Mg/Al hydrotalcite is that they behave as solid Brønsted bases, the active base sites being mainly structural hydroxyl anions. The base amount estimated by titration using benzoic acid indicated that samples with different Mg/Al ratios have a small amount of base sites [17]. The calcination process of the Mg/Al hydrotalcite destroys its structure by removing the interlayer water, interlayer carbonates and the hydroxyls. The result is an atomic mixture of Mg/Al mixed oxides phases. The calcination condition is a crucial factor influencing relevant features of the resulting oxides. The thermal treatment must be at a temperature high enough to decompose the interlayer anions, but it cannot exceed a critical temperature, at which the phase segregation and sintering effects take place. Lewis acid sites and Lewis base and Brønsted sites present on the surface of calcined hydrotalcites, were determined through pyridine and CO_2_ adsorption and microcalorimetry with CO_2_ and benzoic acid [18]. Xie and collaborators determined the basicity of calcined Mg/Al hydrotalcite by the Hammett titration method. Calcined hydrotalcites contain surface Brønsted weakly basic OH^−^ groups, Lewis medium (Mg–O pairs) and strong basic sites related to isolated O^−2^ anions [19]. It has been shown that microwave radiation assisted the esterification of free fatty acid, the synthesis of acylals and anhydro-dimers of *o*-hydroxybenzaldehydes and regioselective ring opening of oxiranes with amines using sulphated zirconia as catalyst [20,21,22].

PCU-like structures have been used as hosts for chiral ammonium ion, as binders in the enantioselective alkylation of benzaldehyde with diethylzinc [23], in enantioselective Michael reaction [24] and recently in the synthesis of diamine derivatives, which are potential anti-inflammatory agents used in the treatment of arthritis rheumatoid [25]. Cyanohydrins are important building blocks for the synthesis of α-hydroxy acids, α-amino acids, α-hydroxy aldehydes or ketones, β-amino alcohols, and vicinal diols. Enantioselective cyanosilylation of carbonyl compounds is an effective reaction of nucleophilic addition in the synthesis of optically active cyanohydrins [26]. Hence, their application would be key to functionalization of some structures, in this particular case the pentacyclic [5.4.0.0^2,6^.0^3,10^.0^5,9^] undecane-8,11-dione.

Most organic synthesis reactions, had only been performed using homogeneous acid or basic catalysis, however, performing reactions under these conditions has disadvantages in terms of pollution problems due to the purification processes and the use of solvents during their development. For such a reason and in order to avoid unwanted situations, in recent years it has been proposed the use of acid [27] and basic [28] heterogeneous catalysts, which make it possible to reduce the use of corrosive substances, thus promoting the principles of green chemistry [29].

Microwaves are now widely applied in synthesis processes to reduce reaction times and improve yields, these two factors together with the use of solid catalysts in solvent-less conditions facilitate processes like isolation and products purification [30,31,32,33].

**Figure 1:**
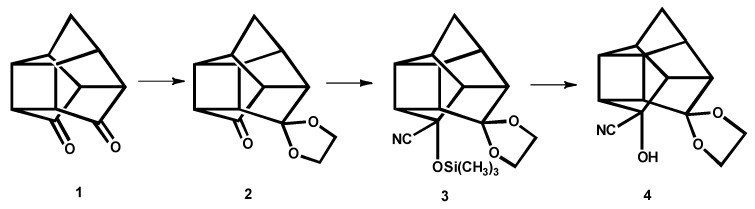
PCU protection, cyanosilylation and deprotection reactions.

In this paper we describe the use of solid catalysts with acid and basic properties: Sulphated zirconia (SZ) and hydrotalcite (HT) respectively, in three types of reactions: (i) Protection of pentacyclic [5.4.0.0^2,6^.0^3,10^.0^5,9^] undecane-8,11-dione **1**, afforded the respective acetal **2**; (ii) The formation of its protected derivative cyanosilylated **3**; and (iii) Subsequent formation of cyanohydrins **4** (Figure 1).

## 2. Results and Discussion

### 2.1. Catalysts Characterization

**Figure 2:**
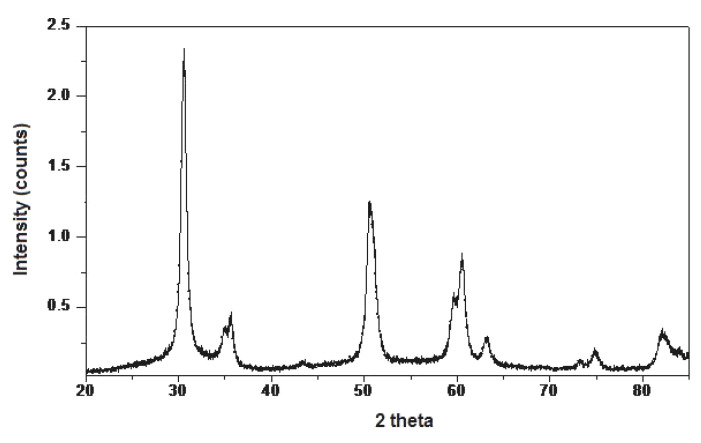
Diffractogram of the sulphated zirconia.

The X-ray powder (XRD) diffractograms of the sulphated zirconia showed the tetragonal phase (ICSD collection code 066787), as given by reflections in 2θ = 30.18° (with 100 as relative intensity) and the reflections located at 34.616°, 35.283°, 43.002°, 50.214°, 50.770°, 59.291°, 60.187°, 62.724°, 74.617° and 81.76° [27] (Figure 2).

**Figure 3:**
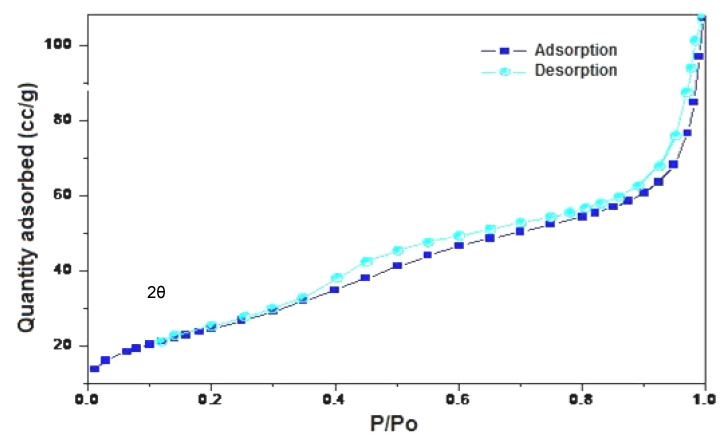
Nitrogen adsorption-desorption plot of sulphated zirconia.

The isotherm obtained through nitrogen physisorption corresponds to type IV IUPAC [34] (Figure 3), which shows a narrow hysteresis loop attributed to capillary condensation in the pores according to their mesopores size [35]. The textural properties calculated were: BET surface area (S_BET_): 90 m^2^/g, the cumulative pore volume (Vp): 0.12 cm^3^/g and the average pore diameter by BJH (Dp): 52.7 Å.

**Figure 4:**
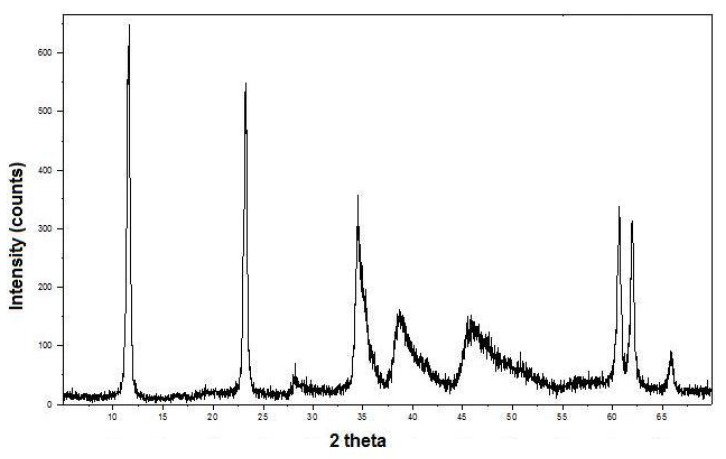
Diffractogram of HTs.

Figure 4 shows the diffractogram of the hydrotalcite bearing hexagonal [36,37,38] symmetry corresponding to the symmetric wide basal planes (003), (006) and (009), while the non-basal planes (102), (105) and (108) are asymmetrical.

**Figure 5:**
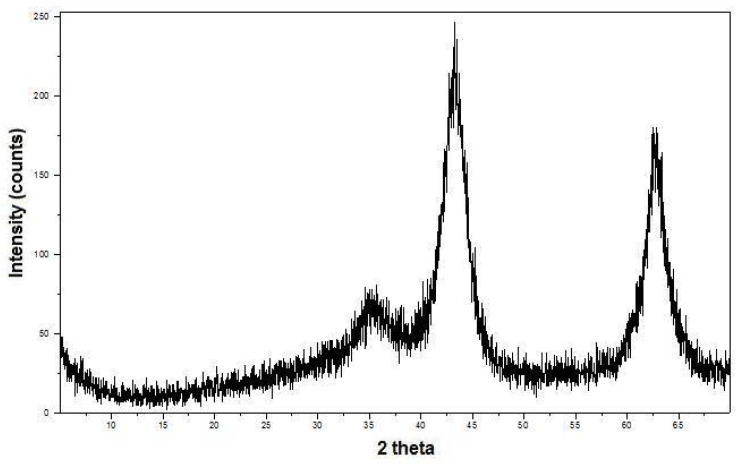
Diffractogram of HTc.

Thermal decomposition of hydrotalcite at 450 °C leads to decomposition of carbonate and dehydroxylation, obtaining the mixed oxide Mg(Al)O, with a structure similar to that of MgO, periclase-type [38]. In the periclase phase, the peaks observed correspond to (200) and (220) planes, with angles centered at 2θ = 43° and 62°, respectively (Figure 5).

**Figure 6:**
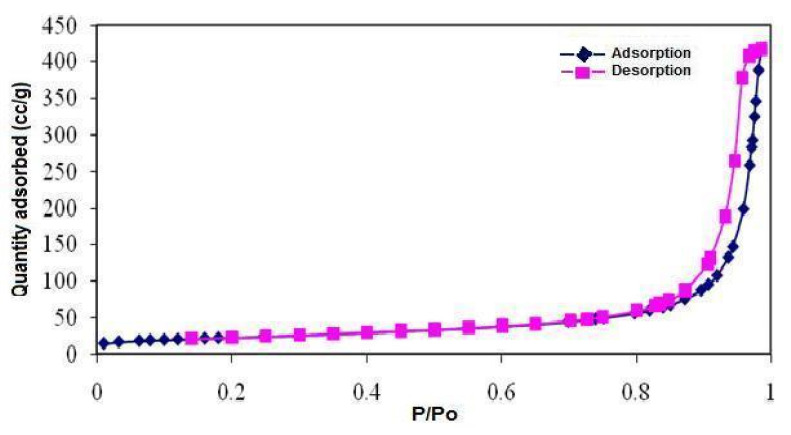
Nitrogen adsorption-desorption plot of HTs.

**Figure 7:**
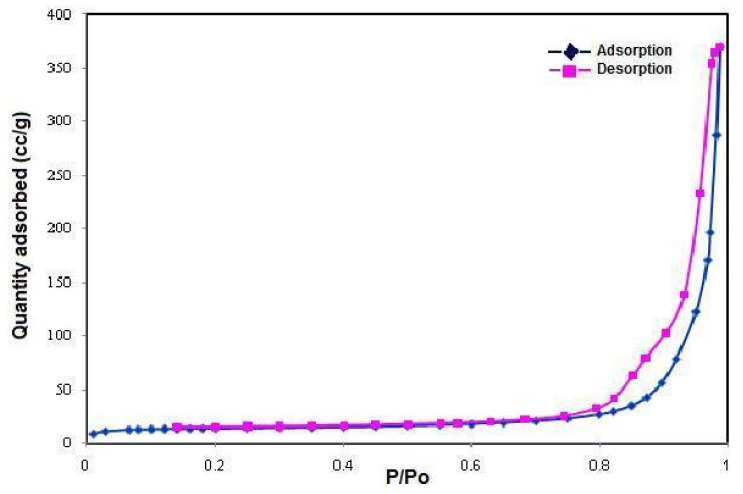
Nitrogen adsorption-desorption plot of HTc.

Textural properties of the hydrotalcites prepared are summarized in Table 1. After thermal decomposition by calcination the porosity increases substantially generating a more pronounced hysteresis loop in the isotherm of the calcined hydrotalcite (HTc), indicating the presence of mesoporosity. The adsorption-desorption isotherms of N_2_ of the hydrotalcites are shown in Figure 6 and Figure 7 and corresponds to Type II IUPAC, and a hysteresis loop type H3 under the same classification.

**Figure 8:**
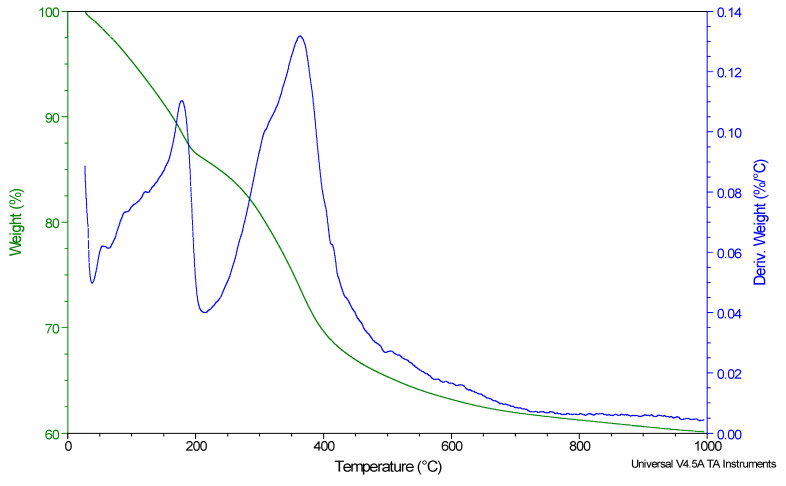
TGA and DTA profiles of HTs.

**Figure 9:**
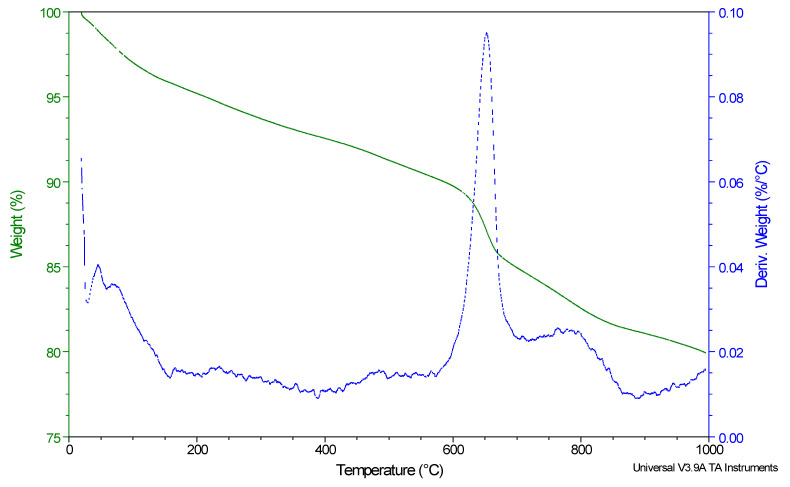
TGA and DTA profiles of sulphated zirconia.

The thermal stability of the as-synthesized and calcined hydrotalcite were evaluated by TGA experiments. Two mayor weight loss peaks are observed in Figure 8. The first peak (near 177 °C) has been asociated with elimination of physically adsorbed water as well as water molecules in the interlamellar region 11.63%. The second weight loss peak (around 364 °C) was due to removal of interlayer anions and the deshydroxylation of layer hydroxyl groups 26.29% [39]. Sulphated zirconia losses only 1% of weight due to water before 100 °C and then 10% after 600 °C due to sulphate species decomposition (Figure 9). The basic character of as-synthezised and calcined hydrotalcites were compared by CO_2_ adsorption FT-IR experiments. CO_2_ was adsorbed on a sample of as-synthezised hydrotalcite, then desorbed at 5, 20, 50, 100, 200, 300, 400, and 450 °C and did not show temperature dependence. On the other hand, the calcined hydrotalcite displayed a decrease of the CO_2_ desorbed quantity as a function of increasing temperature. Deconvolution analysis of FT-IR spectra in the region of 1200–1800 cm^−1^ showed that the calcined hydrotalcite basic catalytic activity is due to the CO_2_ interaction with basic sites with aprotic character on the structure of the solid material. This is explained by the absence of the bicarbonate band.

**Figure 10:**
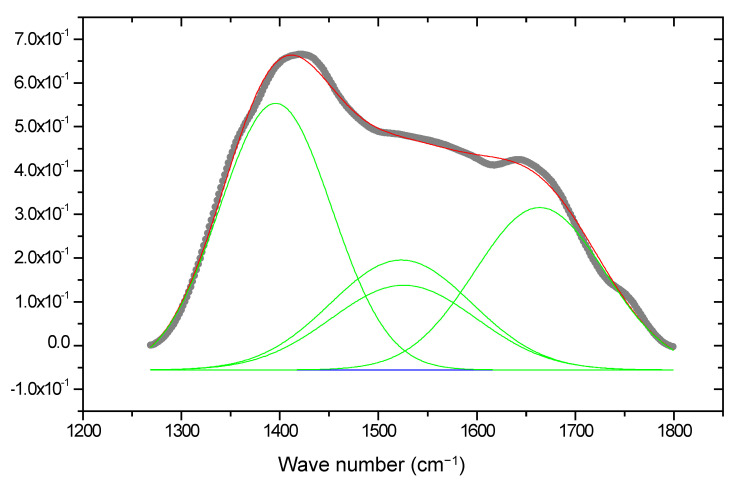
Deconvolution plot of the carbonates zone FT-IR spectra of as-synthezised hydrotalcite.

**Figure 11:**
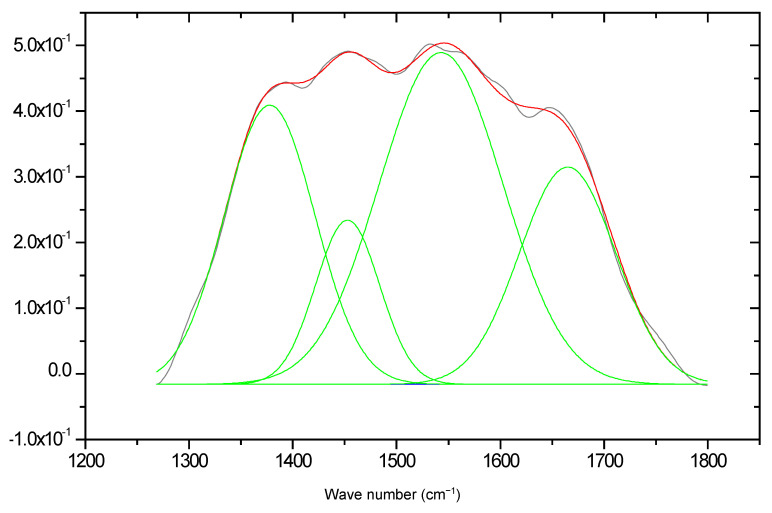
Deconvolution plot of carbonates zone FT-IR spectra of calcined hydrotalcite.

Figure 10 and Figure 11 correspond to deconvolution analysis of the carbonates region for both hydrotalcites; the plots demonstrate a variation on the distribution of the carbonate species on as-synthezised and calcined samples; as-synthezised hydrotalcite had 37% of monodentate carbonates and 62% bidentate species. The calcined showed 26% of monodentate carbonates and 74% of bidentated [40].

### 2.2. Catalytic Activity

**Scheme 1:**
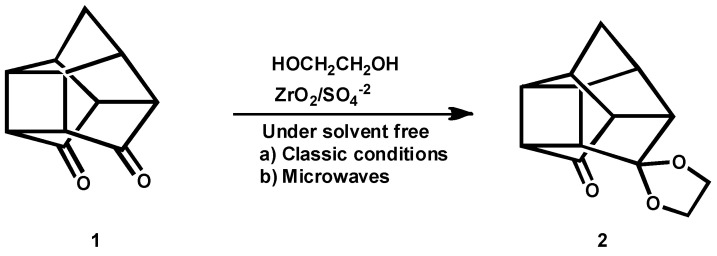
Protection reaction.

Microwave irradiation-assisted pentacyclo [5.4.0.0^2,6^.0^3,10^.0^5,9^] (PCU) **1** transformation coupled with solvent-less reaction medium conditions, in the presence of heterogeneous catalysts (sulfated zirconia and calcined hydrotalcite Mg/Al) were used to enhance reaction rates and to simplify work-up, particularly purification. The preparation of octahydrospiro[2,4,1(epiethane[1,1,2]triyl)cyclobuta [cd]pentalene-5,2'-[1,3]dioxolan]-7-one (**2**), a typical acid-catalyzed reaction, could be conducted successfully with very good yield (99%) in only five minutes. In classic thermal conditions, at 80 °C using toluene as solvent, the reaction time was 45 minutes, Scheme 1.

**Scheme 2:**
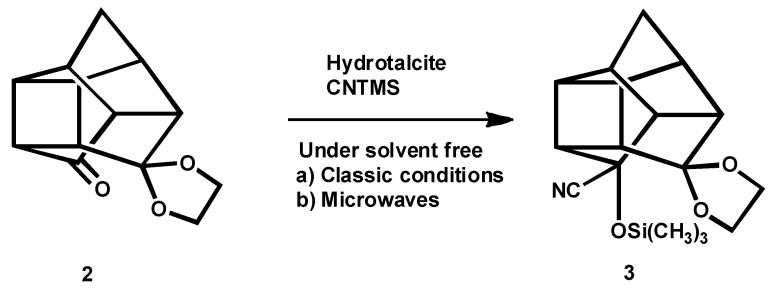
Cyanosilylation reaction.

A simple and convenient method for the addition of TMSCN to protect the PCU compound **2** is also described. Calcined hydrotalcite Mg/Al under solvent-free conditions at 0 °C and microwave-assisted is found to possess a strong basic property to transform **2** smoothly into the corresponding 7-hydroxyoctahydrospiro[2,4,1-(epiethane[1,1,2]triyl)cyclobuta[cd]pentalene-5,2'-[1,3]dioxolane]-7-carbonitrile **3** in high yields (94% in 1.5 h). In classic thermal conditions at 0 °C, the reaction time was 5 h with 98% yield, Scheme 2.

Sulphated zirconia is found to be a highly efficient heterogeneous solid catalyst in the activation reaction of trimethylsilyl cyanide (TMSCN) to facilitate aldehyde cyanosilylation. The sulphated zirconia is a chemoselective catalyst for the cyanosilylation reaction of aldehydes [41], while hydrotalcites catalyze both aldehydes and ketones [42].

**Scheme 3:**
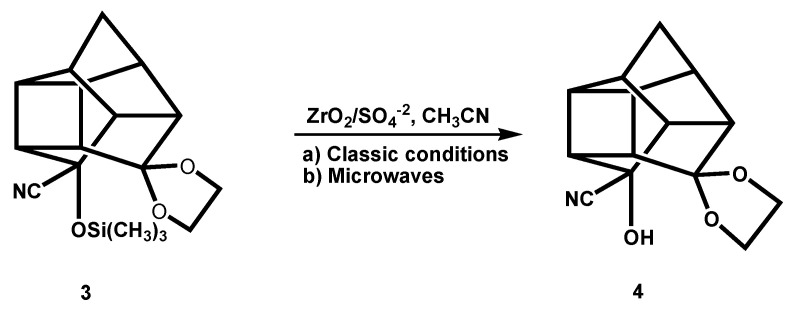
Deprotection reaction.

The microwave-assisted deprotection reaction of cyanosilylated compound **3** was carried out in acetonitrile solvent using sulphated zirconia as catalyst, giving 7-((trimethylsilyl)oxy)octahydrospiro[2,4,1-(epiethane[1,1,2]triyl)cyclobuta[cd]pentalene-5,2'-[1,3]dioxolane]-7-carbonitrile, Scheme 3.

Table 2 summarizes the optimal reaction times and yields of each product obtained from protection-cyanosilylation-deprotection reactions of pentacyclic [5.4.0.0^2,6^.0^3,10^.0^5,9^]undecane-8,11-dione.

Under the reaction conditions described above, in each case the conversion of the starting material is close to 100%, with the selective formation of products labeled in (Figure 1) as **2**, **3** and **4** respectively, which implies a high efficiency of the reaction procedure thus described, to obtain the cyanohydrin derived from the pentacyclic [5.4.0.0^2,6^.0^3,1^0.0^5,9^]undecane-8,11-dione (PCU). As can be seen through comparison of the yields obtained by conventionally heated methods, the use of microwaves in the case of protective reactions of the PCU, gave yields that were almost the same, although with the use of microwave reaction only the time improves, whereas for subsequent reactions, from cyanosilylation and deprotection, the use of microwave radiation improves both the yields and reaction times.

The combined merits of microwave irradiation solvent free condition and solid catalysts make the organic transformation a safe operation, reducing pollution to low levels, giving rapid access to products and simplifying workup. The solid catalysts sulphated zirconia and calcined hydrotalcite Mg/Al can be recovered by centrifugation, reactivated through thermal treatment and reused in at least five reaction cycles.

## 3. Experimental

### 3.1. General

*Sulphated zirconia synthesis.* Zirconium *n*-propoxide (20 mL, 70% *n*-propanol) was mixed with 2-propanol (30 mL) and stirred with a magnetic bar. Then, an acid solution (1 mL 98% sulfuric acid in 3.2 mL distilled water) was added dropwise in order to hydrolyze the zirconium *n*-propoxide to obtain the gel. The solid was filtered and dried at 80 °C until complete alcohol evaporation, then calcined in air at 600 °C for 6 h [43].

*Hydrotalcite synthesis.* A solution of NaOH (0.35 mol) and Na_2_CO_3_ (0.093 mol) in 100 mL of deionized water was added to a solution of Mg(NO_3_)·6 H_2_O (0.1 mol) and Al(NO_3_)_3_·9H_2_O (0.05 mol) in 70 mL of deionized water. The addition was made dropwise over 4 h, thus forming a white gel which is then stirred and heated to 60 °C for 18 h. The resulting gel was allowed to cool and washed with deionized H_2_O to pH = 7. The compound was dried at 100 °C overnight, thereby obtaining the dry hydrotalcite (HTs). The calcined hydrotalcite (HTc) was obtained by heating 2 g of HTs 10 °C/min to 450 °C in a tubular furnace (Thermolyne 21100) under air flow for 8 h [28].

Powder X ray diffraction (XRD) was performed with a Philips X’Pert Instrument using Cu-Kα radiation (45 kV, 40 mA). Nitrogen adsorption/desorption isotherms were obtained at −196 °C on Micromeritics ASAP 2020 equipment. The reaction products were analyzed by GC-FID (Agilent Technologies 6890N) equipped with a HP-5 column with the program 70–180 °C (20 °C /min) for 6.50 min then 180–280 °C (15 °C/min) for 7 min, injector 250 °C, detector 280 °C. Mass spectra were obtained by GC-MS (Agilent Technologies 6890N, Detector 5973) using methane chemical ionization. ^1^H-NMR and ^13^C-NMR spectra were measured at 500 MHz and 125 MHz, respectively, with the aid of a Bruker Avance-III DMX-500 spectrometer, using CDCl_3_ as solvent and tetramethylsilane as internal standard. FT-IR spectra were recorded also with a Bruker Vector 33 ATR spectrometer. All reagents and solvents were purchased from Aldrich. All products were identified by IR, ^1^H-NMR, ^13^C-NMR, mass spectra and by comparison of their corresponding melting points. The hydrotalcites’ basicity was measured using FT-IR with CO_2_ (research grade) as probe molecule. Measurements were performed using a Nicolet Magna IR 560 spectrometer equipped with a Harrick ATC Prayin Mantis accessory. The as synthesized and calcined hydrotalcite samples were treated from room temperature to 500 °C under nitrogen atmosphere (20 mL/min) then cooled, CO_2_ was introduced to the chamber during 1 h (20 mL/min) and finally, the temperature was increased to 500 °C while the FT-IR spectra were recorded. TGA and DTA analysis of hydrotalcites were made using a TA SDT Q600 equipment. A 10 °C/min ramp under nitrogen was used from room temperature to 1000 °C.

### 3.2. Procedure for the PCU Protection

*Conventional heating method.* In a closed system were placed PCU (**1**, 1.15 mmol), ethylene glycol (1.15 mmol) and toluene as solvent (1 mL) in the presence of sulphated zirconia (25 mg). The reaction mixture was stirred for 45 min at 80 °C, the sulphated zirconia was recovered by filtration and washed with CH_2_Cl_2_. The filtrate was dried with anhydrous sodium sulfate and evaporated to dryness, obtaining the corresponding pentacyclic [5.4.0.0^2,6^.0^3,10^.0^5,9^]undecane-8,11-dione monoethylene acetal **2** (yield 99.8%).

*Microwave assisted method.* In a closed system were placed: PCU (**1**, 1.0 mmol) and ethylene glycol (1 mL) without solvent, and with sulphated zirconia (25 mg). The reaction mixture was placed in a CEM Discover Labmate® reactor at 150 °C and 290 W for 5 min with rapid stirring. Subsequently, the reaction crude was filtered, evaporated to dryness and purified on a chromatographic column to give selectively the corresponding pentacyclic [5.4.0.0^2,6^.0^3,10^.0^5,9^]undecane-8,11-dione monoethylene acetal **2** (yield 99.7%).

*Octahydrospiro[2,4,1-(epiethane[1,1,2]triyl)cyclobuta[cd]pentalene-5,2'-[1,3]dioxolan]-7-one* (**2**). White solid (yield 99.7%); m.p. 69–70 °C. (m.p. lit. 73–73.5 °C [28]); GC-MS for C_13_H_14_O_3_ (218 g/mol): [M+1]^+^ = 219, [M+29]^+^ = 247 y [M+41]^+^ = 259; FTIR-ATR (cm^−1^): 2979, 1742, 1340, 1121, 1094, 1039; ^1^H-NMR (500 MHz; CDl_3_): δ (ppm) 3.98–3.91 (m, 3H), 3.90–3.84 (m, 1H), 2.99–2.95 (m, 1H), 2.85–2.80 (m, 2H), 2.66 (dddd, *J* = 8.2, 6.6, 3.0, 1.4 Hz, 1H), 2.62 (ddq, *J* = 5.4, 4.1, 1.4 Hz, 1H), 2.57 (dddd, *J* = 8.4, 6.2, 2.3, 1.1 Hz, 1H), 2.52–2.49 (m, 1H), 2.44 (ddt, *J* = 10.0, 4.1, 2.0 Hz, 1H), 1.88 (dt, *J* = 11.0, 1.5 Hz, 1H), 1.59 (dt, *J* = 11.0, 1.5 Hz, 1H); ^13^C-NMR (125 MHz; CDl_3_): δ (ppm) 215.02, 113.79, 65.58, 64.38, 52.89, 50.61, 45.72, 42.75, 42.17, 41.36, 41.21, 38.60, 36.22.

### 3.3. Procedure for the Cyanosilylation of Pentacyclic [5.4.0.0^2,6^.0^3,10^.0^5,9^]undecane-8,11-dione Mono-ethylene Acetal *(**2**)*

*Conventional heating method.* In a closed system, there were placed pentacyclic [5.4.0.0^2,6^.0^3,10^.0^5,9^]undecane-8,11-dione monoethylene acetal (**2**, 1.0 mmol) and TMSCN (2.0 mmol) without solvent, and calcined hydrotalcite (25 mg) and the reaction mixture was stirred for 5 h. Subsequently, the catalyst was removed by filtration and the liquid obtained was evaporated to dryness and purified on a chromatographic column to give selectively the desired new cyanohydrin **3** (yield 88.8%).

*Microwave-assisted method.* In a closed system there were placed pentacyclic [5.4.0.0^2,6^.0^3,10^.0^5,9^]undecane-8,11-dione monoethylene acetal (**2**, 0.5 mmol) and TMSCN (1.0 mmol) without solvent, and calcined hydrotalcite (25 mg) and the reaction mixture was placed in a CEM Discover Labmate® microwave reactor at 0 °C and 290 W, with rapid stirring for 1.5 h. Subsequently, the catalyst was removed by filtration and the liquid obtained was evaporated to dryness and purified on a chromatographic column to give selectively the desired cyanohydrin **3** (yield 94.1%).

*((7-Isocyanooctahydrospiro[2,4,1-(epiethane[1,1,2]triyl)cyclobuta[cd]pentalene5,2'[1,3]dioxolan]-7-yl)oxy)trimethylsilane* (**3**). Yellow solid (yield 94.1%). m.p. 94–95 °C; GC-MS for C_17_H_23_NO_3_Si; (317 g/mol): [M+1]^+^ = 318, [M+29]^+^ = 346, [M−CN]^+^ = 291; FTIR-ATR (cm^−^^1^): 2955, 2240, 1307, 1251, 1102, 880, 838, 782; FAB-MS *m/z* (rel. int.%): obs. 317.1422 (55.2) cal. 317.1447; ^1^H-NMR (CDCl_3_, 500 MHz): δ (ppm) 3.94–3.91 (m, 2H), 3.83–3.79 (m, 1H), 3.74 (ddd, *J* = 7.6, 6.7, 6.4 Hz, 1H), 3.02–2.99 (m, 1H), 2.83 (tq, *J* = 4.7, 1.6 Hz, 1H), 2.71–2.65 (m, 4H), 2.50–2.46 (m, 1H), 2.23–2.20 (m, 1H), 1.70 (d, *J* = 11.1 Hz, 1H), 1.25 (dt, *J* = 11.0, 1.7 Hz, 1H), 0.24 (s, 9H); ^13^C-NMR (125 MHz; CDl_3_): δ (ppm) 123.10, 114.40, 75.15, 65.96, 62.42, 51.56, 47.42, 44.96, 44.75, 43.91, 39.79, 39.59, 38.60, 34.46, 0.86.

### 3.4. Deprotection Procedure for the Pentacyclic [5.4.0.0^2,6^.0^3,1^0.0^5,9^]undecane-8,11-dione Mono-ethylene Acetal Cyanosilylated ***3***

*Conventional heating method.* In a closed system the cyanohydron of the pentacyclic [5.4.0.0^2,6^.0^3,10^.0^5,9^]undecane-8,11-dione monoethylene acetal (1.0 mmol) and acetonitrile (1.0 mL) were placed with sulphated zirconia (25 mg). The reaction mixture was stirred for 6 h at 60 °C. Subsequently, the catalyst was filtered selectively obtaining the desired new product **4** (yield 90.1%).

*Microwave-assisted*. In a closed system, there were placed the product cyanohydrin of the pentacyclic [5.4.0.0^2,6^.0^3,10^.0^5,9^]undecane-8,11-dione monoethylene acetal 3 (1.0 mmol) and acetonitrile (1.0 mL) in the presence of sulphated zirconia (25 mg). The reaction mixture was stirred in a CEM Discover Labmate® microwave reactor at 60 °C and 100 W for 20 min, with rapid stirring. Subsequently the catalyst was filtered selectively obtaining the desired new product **4** (yield 91.7%).

*7-Isocyanooctahydrospir*o*[2,4,1-(epiethane[1,1,2]triyl)cyclobuta[cd]pentalene-5,2'-[1,3]dioxolan]-7-ol* (**4**). Yellow oil. GC-MS for C_14_H_15_NO_3_ (m.w.: 245 g/mol): [M+1]^+^ = 246, [M+29]^+^ = 274, [M+41]^+^ = 286; FTIR-ATR (cm^−^^1^): 3433, 2974, 2871, 2247, 1342, 1275, 1212, 1143, 1036, 904, 868, 644; FAB-MS *m/z* (rel. int.%): Obs. 245.0989 (66.3) calc. 245.1052; ^1^H-NMR (500 MHz, CDCl_3_): δ (ppm), 3.82–3.80 (m, 2H), 3.78–3.75 (m, 2H), 3.26–3.22 (m, 1H), 3.21–3.17 (m, 1H), 2.87–2.79 (m, 4H), 2.75–2.72 (m, 1H), 2.64–2.61 (m, 1H), 2.13–2.11 (m, 1H), 1.99 (dt, *J* = 10.9, 1.5 Hz, 1H), 1.64 (dt, *J* = 11.0, 1.6 Hz, 1H); ^13^C-NMR (125 MHz, CDCl_3_): δ (ppm) 122.63, 117.96, 78.44, 68.15, 61.94, 60.63, 53.70, 50.83, 45.38, 43.58, 43.38, 42,76, 42.23, 41.25.

## 4. Conclusions

We have demonstrated that the use of sulphated zirconia and hydrotalcites Mg/Al, provides efficient results in the organic transformations as protection, cyanosililation and deprotection of the pentacyclo [5.4.0.0^2,6^.0^3,10^.0^5,9^]undecane-8,11-dione, under soft treatment and in the absence of solvents. Microwave-assisted method improves the performances that are obtained under thermal classic conditions and diminishing the reaction times.

## Figures and Tables

**Table 1:** Hydrotalcites’ textural properties.

Catalyst	Surface area (S_BET_) (m^2^/g)	Pore Volume (cc/g)	Pore diameter (Å)
Hts	66.62	0.44	266.88
Htc	241.21	0.74	159.70

**Table 2:** Catalytic activity results.

Substrate	Time(h)	Product	Yield (%)
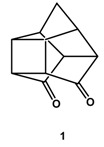	0.75 ^a^ 0.083 ^b^	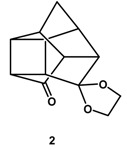	99.8 ^a^ 99.7 ^b^
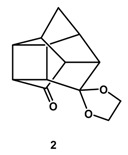	5.00 ^c^ 1.30 ^d^	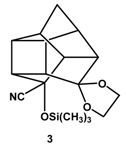	88.8 ^c^ 94.1 ^d^
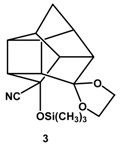	6.00 ^e^ 0.33 ^f^	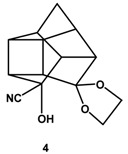	90.1 ^e^ 91.7 ^f^

*Reaction conditions*: (a) PCU (1.15 mmol), ethylene glycol (1.15 mmol), toluene (1 mL), ZrO_2_/SO_4_^−2^ (25 mg), 80 °C; (b) PCU (1 mmol), ethylene glycol (1 mL), solventless, ZrO_2_/SO_4_^−2^ (25 mg), 150 °C, 290 Watts; (c) Pentacyclic [5.4.0.0^2,6^.0^3,1^0.0^5,9^]undecane-8,11-dione monoethylene-acetal (2 mmol), TMSCN (2 mmol), solventless, calcined hydrotalcite (25 mg), 0 °C; (d) Pentacyclic [5.4.0.0^2,6^.0^3,1^0.0^5,9^] undecane-8,11-dione monoethylene-acetal (2 mmol), TMSCN (2 mmol), solventless, calcined hydrotalcite (25 mg), 0 °C, 290 Watts; (e) Pentacyclic [5.4.0.0^2,6^.0^3,1^0.0^5,9^]undecane-8,11-dione monoethylene-acetal cyanosilylated (1 mmol), acetonitrile (1 mL), ZrO_2_/SO_4_^−2^ (25 mg), 60 °C; Pentacyclic [5.4.0.0^2,6^.0^3,1^0.0^5,9^]undecane-8,11-dione monoethylene-acetal cyanosilylated (1 mmol), acetonitrile (1 mL), ZrO_2_/SO_4_^−2^ (25 mg), were irradiated at 60 °C, 20 min, 100 Watts.
